# Changes in Body Composition during Adjuvant FOLFOX Chemotherapy and Overall Survival in Non-Metastatic Colon Cancer

**DOI:** 10.3390/cancers12010060

**Published:** 2019-12-24

**Authors:** Eric Chung, Hye Sun Lee, Eun-Suk Cho, Eun Jung Park, Seung Hyuk Baik, Kang Young Lee, Jeonghyun Kang

**Affiliations:** 1 Yonsei University College of Medicine, Seoul 03722, Korea; erichyunchung@gmail.com; 2 Biostatistics Collaboration Unit, Yonsei University College of Medicine, Seoul 06273, Korea; HSLEE1@yuhs.ac; 3 Department of Radiology, Gangnam Severance Hospital, Yonsei University College of Medicine, Seoul 06273, Korea; JJONDOL@yuhs.ac; 4 Department of Surgery, Gangnam Severance Hospital, Yonsei University College of Medicine, Seoul 06273, Korea; camp79@yuhs.ac (E.J.P.); whitenoja@yuhs.ac (S.H.B.); 5 Department of Surgery, Severance Hospital, Yonsei University College of Medicine, Seoul 03722, Korea; kylee117@yuhs.ac

**Keywords:** colon cancer, skeletal muscle, visceral fat, subcutaneous fat, radiodensity, overall survival

## Abstract

The impact of longitudinal anthropometric changes during adjuvant chemotherapy on long-term survival in non-metastatic colon cancer is unclear. Herein, we analyzed the prognostic significance of computed tomography (CT)-measured body composition changes in colon cancer patients who underwent surgery followed by adjuvant FOLFOX (folinic acid, 5-fluorouracil, oxaliplatin) chemotherapy. Data of 167 patients with stage III or high-risk stage II colon cancer were analyzed. Skeletal muscle index (SMI), skeletal muscle radiodensity (SMR), visceral fat index (VFI), subcutaneous fat index (SFI), and total fat index (TFI) changes during chemotherapy were calculated using preoperative and postchemotherapy CT image data. The Cox proportional hazard model was used to determine the correlation between changes in anthropometric values and overall survival (OS). The median changes (%) in SMI, SMR, VFI, SFI, and TFI over 210 days during chemotherapy were 8.7% (*p* < 0.001), 3.4% (*p* = 0.001), −19% (*p* < 0.001), −3.4% (*p* = 0.936), and −11.9% (*p* < 0.001), respectively. Cut-off values of changes in SMI (skeletal muscle index change, SMIC) and SMR (skeletal muscle radiodensity change, SMRC) were defined at −2% and −2 Hounsfield units (HU) respectively, whereas those of changes in VFI (visceral fat index change, VFIC), SFI (subcutaneous fat index change, SFIC), and TFI (total fat index change, TFIC) were based on values that provided the largest χ2 on the Mantel–Cox test. Multivariable analysis revealed that low SMR measured on a postchemotherapy CT scan (hazard ratio, HR: 0.32, 95% confidence interval, CI: 0.15–0.70, *p* = 0.004) and visceral fat loss of at least 46.57% (HR: 0.31, 95% CI: 0.14–0.69, *p* = 0.004) were independent poor prognostic factors for OS. Severe visceral fat loss during FOLFOX chemotherapy and low skeletal muscle radiodensity measured on postchemotherapy CT scans are associated with poor OS in stage III and high-risk stage II colon cancer patients.

## 1. Introduction

Colorectal cancer (CRC) is one of the leading causes of cancer-related deaths worldwide [1]. Curative resection is the treatment of choice, and adjuvant chemotherapy is recommended for potentially increasing survival in colon cancer patients diagnosed with stage III and stage II with high-risk factors [2]. Although various chemotherapy regimens have been recommended in these cases, FOLFOX (folinic acid, 5-fluorouracil, and oxaliplatin) is one of the most commonly used adjuvant chemotherapy regimens, with clear indications for use in the postoperative setting [3,4,5]. However, well-described risk factors to predict mortality after completion of chemotherapy are lacking in these relatively high-risk groups; therefore, recommendations for identifying ideal candidates for more intensive follow-up have not been characterized.

The impact of skeletal muscle depletion (known as sarcopenia) or decreased skeletal muscle radiodensity (known as myosteatosis) has been investigated in various cancers with respect to chemotherapy- or chemoradiotherapy-induced toxicities or survival [6,7,8,9,10]. Sarcopenia, myosteatosis, and visceral obesity have demonstrated substantial value as prognosticators in a number of studies on various cancers including CRC [11,12,13]. In addition to cross-sectional data-driven analysis, several studies have investigated the impact of skeletal muscle changes during treatment in patients with CRC. Skeletal muscle loss during chemotherapy is known to be associated with poorer survival and is known to predict an increased risk of dose-limiting toxicities, particularly in patients with metastatic or advanced CRC [14,15,16]. Previous studies have investigated the prognostic significance of longitudinal changes in skeletal muscle index (SMI), skeletal muscle radiodensity (SMR), and visceral fat index (VFI) in patients with non-metastatic CRC, using data from baseline and follow-up computed tomography (CT) scans. However, in previous studies, the time span between the two CT scans was variable, with durations of 9–27 months, 24 months, and more than 1 year after surgery or adjuvant chemotherapy; in addition, patients with early recurrence were not included in certain studies [17,18,19]. Therefore, the clinical applicability of these results may be limited in patients with longer survival, who undergo long-term follow-up CT examinations without recurrences. To date, studies simultaneously analyzing the impact of longitudinal changes in muscle and fat in this setting are lacking.

Therefore, this study aimed to investigate the prognostic significance of longitudinal changes in body composition including muscle and fat during adjuvant FOLFOX chemotherapy in patients with stage III and high-risk stage II colon cancer.

## 2. Results

We identified a total of 214 patients with colon cancer who were treated with adjuvant FOLFOX chemotherapy after surgery. Five patients were excluded owing to administration of neoadjuvant chemotherapy (n = 3) and a history of inflammatory bowel disease (n = 2); thirty-three patients were excluded owing to missing data or the sub-optimal quality of preoperative CT (n = 9) or postchemotherapy CT scans (n = 24). Among those who underwent CT measurements, patients with intestinal obstruction showing on their preoperative CT scans (n = 9) were excluded as data on correct measurements of visceral fat were limited in these cases. Among the 214 patients, 167 had met the defined criteria and constituted our study sample. Among the included patients, 137 patients (82%) completed 12 cycles of chemotherapy, and 95% of patients underwent more than six cycles of FOLFOX chemotherapy (Supplementary Figure S1).

The baseline characteristics of the patients, based on their gender, are presented in Table 1. Among 167 patients, 67 were female and 100 were male. There were no differences in clinicopathological variables between female and male subgroups, except body mass index (BMI). The proportion of patients who had a BMI exceeding 25 kg/m^2^ and who were smokers was significantly higher in the male than in the female subgroup (34% vs. 17.9%, *p* = 0.035 and 47% vs. 0%, *p* < 0.001, respectively).

Absolute changes in body composition during FOLFOX adjuvant chemotherapy were evaluated according to each variable. SMI and SMR increased (*p* < 0.001 and *p* = 0.001, respectively), and VFI decreased significantly (*p* < 0.001); however, there was no change in SFI (*p* = 0.936) (Supplementary Tables S1 and S2). Anthropometric values measured using preoperative and postchemotherapy CT scans and their changes over 210 days were compared between patients from either gender. The mean values of SMI_pre, SMR_pre, and VFI_pre were significantly higher in the male than the female subgroup, whereas SFI_pre was significantly higher in the female subgroup. Except for TFI, these trends were maintained in data extracted from postchemotherapy CT scans. The mean value of TFI_post was significantly higher on postchemotherapy CT; however, there was no difference on the preoperative CT. Except for SMIC, there was no difference between genders in terms of percentage changes over 210 days. The SMIC over 210 days was significantly higher in the female than the male subgroup (Table 2).

**Figure 1:**
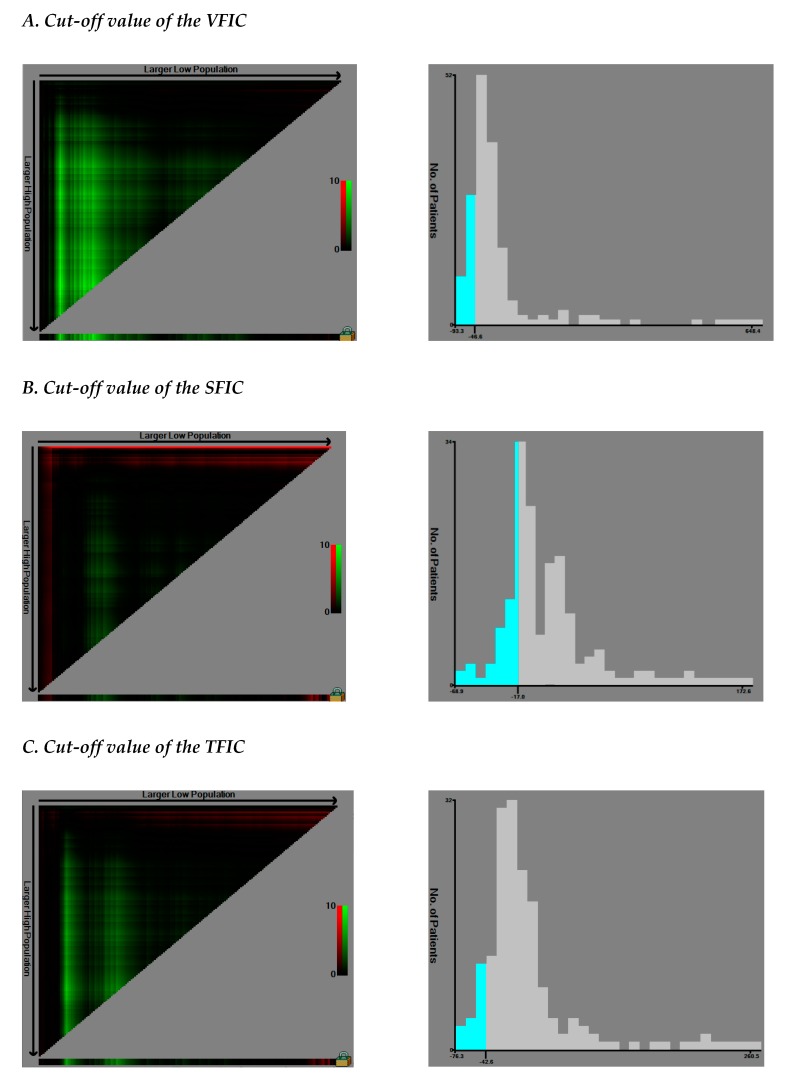
Determining cut-off values of visceral fat index change (VFIC), subcutaneous fat index change (SFIC), and total fat index change (TFIC) using an X-tile program (*n* = 167). X-tile plots of the VFIC (**A**), SFIC (**B**), and TFIC (**C**) and the points of the variable coloration of the plot represent the strength of the association at each division ranging from low (dark, black) to high (bright, red or green). Red represents an inverse association between the expression levels and survival of the variables, whereas green represents a direct association. The optimal cut-off values were defined as the values that produced the largest χ2 in the Mantel–Cox test, and these points were set as −46.57 (VFIC) (**A**), −17.03 (SFIC) (**B**), and −42.61 (TFIC) (**C**), respectively. Patients were divided into the two subgroups based on these values on the following survival analysis.

The median changes (%) in SMI, SMR, VFI, SFI, and TFI over 210 days during chemotherapy were 8.7% (*p* < 0.001), 3.4% (*p* = 0.001), −19% (*p* < 0.001), −3.4% (*p* = 0.936), and −11.9% (*p* < 0.001), respectively (Table 3, Figure S2). The cut-off values of SMIC and SMRC were defined at −2%, respectively, whereas those of VFIC, SFIC, and TFIC were set at −46.57%, −17.03%, and −42.61%, respectively (Figure 1). Patients were divided into two subgroups during survival analysis based on these values.

On univariable analysis, duration of surgery, lymphovascular invasion (LVI), VFIC, and TFIC were identified as significant predictors of OS (all *p* < 0.05). In addition, smoking, chemotherapy cycles, SMR_pre, SMR_post, and SFIC were found to be associated with OS; however, these did not reach statistical significance (all *p* < 0.1) (Table 4 and Table 5, Supplementary Figures S3–S5). Variables with *p* < 0.1 on univariable analysis were entered for multivariable analysis. Low SMR measured on postchemotherapy CT (HR: 0.32, 95% CI: 0.15–0.70, *p* = 0.004) and visceral fat loss of at least 46.57% during FOLFOX chemotherapy (HR: 0.31, 95% CI: 0.14–0.69, *p* = 0.004) were found to be independent poor prognostic factors for OS in multivariable analysis. In addition, smoking (HR: 2.25, 95% CI: 1.06–4.77, *p* = 0.034), a duration of surgery exceeding 300 min (HR: 3.87, 95% CI: 1.80–8.33, *p* = 0.0005), and the presence of LVI (HR: 2.46, CI: 1.16–5.18, *p* = 0.017) were associated with poorer OS (Table 6).

## 3. Discussion

Our study evaluated the clinical significance of body composition measured at different time points during treatment and its longitudinal alterations in estimating survival within a homogeneous cohort of patients with non-metastatic colon cancer, who underwent surgery followed by FOLFOX chemotherapy. Myosteatosis measured on a postchemotherapy CT scan and the loss of at least 46.57% of visceral fat during chemotherapy were identified as independent adverse prognostic factors. Serial measurements of body composition and the evaluation of changes may be beneficial in predicting prognosis in patients with non-metastatic colon cancer. It may therefore be used in modifying follow-up schedules in these patients with relatively high-risk disease. 

Skeletal muscle loss during chemotherapy or chemoradiotherapy has been found to be associated with poorer survival outcomes in various cancers including those of the breast, pancreas, and cervix [8,9,20]. Reports also suggest that muscle loss during treatment is a significant adverse prognosticator in CRC; however, this association was mainly derived from patients with metastatic or advanced stage disease who underwent palliative chemotherapy [14,15,16,21]. In our study, however, muscle loss (defined as a loss of more than 2%/210 days) during FOLFOX chemotherapy was not identified as an independent prognostic factor. This finding is also inconsistent with those of a recent study from our group, which found muscle loss in excess of 4.2%/100 days during preoperative chemoradiotherapy to be a potential poor prognostic factor in patients with non-metastatic locally advanced rectal cancer [11]. All patients in this study had stage II/III colon cancer and had received identical chemotherapy agents in the post-operative recovery phase, after curative resection. The median change in skeletal muscle bulk over 210 days was not negative (8.7%), and only 22 (13.1%) were classified in the skeletal muscle loss group. Although direct comparisons may be inaccurate, the overall muscle loss in our cohort was probably not as severe as that of previous studies, which suggested that muscle loss during treatment was associated with poor prognosis. It is also known that in patients with metastatic CRC, the frequency of muscle loss differs based on the intensity of chemotherapy [21]. In addition, the severity of individual weight or muscle loss may vary according to the stage of cancer cachexia, which is categorized as precachexia, cachexia, and refractory cachexia according to the international consensus [22]. In view of these findings, muscle changes may vary according to the stage of disease, treatment modalities, and host response, and the impact of muscle loss on survival may differ based on different time points in the trajectory of disease. Further research is required to improve the understanding of the actual impact of muscle loss in the postoperative follow-up period in patients with relatively early-stage CRC. 

Previous studies demonstrated that myosteatosis, evidenced by a decrease of skeletal muscle radiodensity, was associated with adverse outcomes in various types of cancers [10,23,24]. Kroenke and colleagues demonstrated that in patients with CRC, low SMR was associated with higher overall and CRC-specific mortality [25]. Hopkins et al. also reported that myosteatosis was independently predictive of OS (HR: 1.53, 95%; CI: 1.19–1.97) [26]. In contrast, several studies have demonstrated no correlation between myosteatosis and survival outcomes in patients with CRC [27,28]. The discordance in results across studies is difficult to explain; however, it may be attributed to differences in cut-off values or methods used to define myosteatosis. In addition, the timing of performing CT scans also varied between the studies; however, most previous studies used CT scans obtained prior to surgery or the initiation of palliative chemotherapy. Our study demonstrated that myosteatosis observed on a postchemotherapy CT scan instead of a preoperative CT scan was associated with adverse outcomes. This suggested that the clinical impact of radiodensity may depend on the time-point; therefore, serial measurements are highly recommended for improving the accuracy of predicting survival. 

Interestingly, most patients in this study lost visceral fat during the period of chemotherapy; after adjusting for other clinicopathologic parameters, severe loss of visceral fat was associated with poor oncologic outcomes. The correlation of visceral fat loss and poor survival outcomes were investigated in detail in patients with pancreatic cancer. In their study, Dalal and colleagues found that visceral fat losses exceeding 12.9% correlated with poor overall survival (HR: 2.06, 95% CI: 1.05–4.03, *p* = 0.034) in patients with inoperable locally advanced pancreatic cancer, who had undergone chemoradiation; they suggested that tumor-induced lipolytic factors and pro-inflammatory cytokines played a major role in adipose tissue wasting [29,30]. In their study, Kays et al. demonstrated that the fat-only loss group had similar oncologic outcomes to the group with concurrent fat and muscle loss. In a study of patients with advanced pancreatic ductal adenocarcinoma who underwent chemotherapy with 5-fluorouracil, leucovorin, irinotecan, and oxaliplatin in the first line, fat-only and concurrent muscle and fat wasting was also found to be independently predictive of poor prognosis compared with no muscle or fat wasting [31]. The authors proposed that the different phenotypes of wasting within a homogenous disease could be related to molecular and genetic differences, which may play particular roles in specific cachexia; therefore, fat and muscle mass wasting could play equivalent roles [31]. In patients with CRC, the clinical impact of fat change at consecutive time points has rarely been investigated. Hopkins and colleagues recently reported that elevated total adiposity was an independent favorable prognosticator for OS (HR: 0.66, 95% CI: 0.46–0.95, *p* = 0.024); however, the prognostic impact of visceral fat changes was not evident [18]. Choe and colleagues found that a reduction in visceral fat during follow-up was associated with poor survival [19]. These findings appear to concur with the results of our study; however, certain factors require consideration. These two studies measuring fat changes in patients with CRC used follow-up CT scans obtained at 2 years after surgery and more than 1 year after surgery or completion of chemotherapy; patients who developed recurrences within the time point of the second follow-up CT were excluded from those analyses in those studies [26,32]. In view of these factors, the results may not be directly applied to clinical decision-making. Although surgery and post-operative chemotherapy may induce catabolic changes in body composition, the impact is not permanent and may be reversed within 1–2 years [33]. The absolute distribution of visceral fat loss in our study may have differed from that of previous studies owing to the different time points of follow-up CT scans. Our study was able to detect the impact of active body changes immediately on completion of chemotherapy; this may have been the strength of this study and may provide a more efficient approach to modulating follow-up schedules. Although the exact underlying mechanism of severe fat loss during chemotherapy could not be identified and CT scanning is not routinely performed immediately after completion of chemotherapy, our findings emphasize the clinical utility of evaluating body composition changes in these relatively high-risk patients in the short term. 

In this study, smoking was identified as an important poor prognostic factor. Cigarette smoking was known to be significantly associated with CRC incidence [34,35]. In addition, a recent meta-analysis reported random-effects HR estimates (95% CI) for all-cause mortality of 1.26 (1.15–1.37) for current smokers compared with never smokers, which indicated that smoking had detrimental effects on survival even after CRC diagnosis [36]. There is some in vitro evidence that smoking could stimulate cell metastatic ability by altering epithelial-mesenchymal transition and influence chemotherapy efficacy by inducing metabolic and epigenetic alterations [37,38]. Smoking showed a strong influence on the natural history of chronic obstructive pulmonary disease (COPD) and smoking related COPD was a significant predictor of more visit to emergency room and hospital readmission in colon cancer patients treated with adjuvant chemotherapy [39,40]. Experimental evidences revealed that smoking could influence skeletal muscle depletion and some muscle metabolism might be impaired in COPD patients [41,42]. Based on these effects of smoking, we believe that the trajectory association among smoking, body composition changes, and chemotherapy tolerance in patients with colon cancer needs to be further investigated. 

In addition, an operation time of more than 300 min was identified as an independent variable associated with poor oncologic outcomes. Unpredictable intraoperative adverse events can result in a delay in operation time; thus, operation time might reflect the complex nature of surgery or technical difficulties. It was reported that technical difficulties during surgery could jeopardize oncologic outcomes in patients with CRC, although the result was based on laparoscopy colorectal resections [43]. Nevertheless, operation time might be multifactorial, and our findings need to be validated with more clinical datasets. 

This study has certain limitations. First, this was a retrospective single-center study with a relatively small sample size. Nevertheless, our cohort comprised a homogeneous group; all patients had undergone curative resection for non-metastatic colon cancer and had received identical adjuvant chemotherapy. Second, the body composition measured on the preoperative CT scan did not demonstrate any clinical significance; this may be attributed to the fact that the declared cut-off for sarcopenia, myosteatosis, and fat obesity used in this study were probably not appropriate for patients with non-metastatic colon cancer and were not predictive of outcomes in the East Asian population. This study could not provide specific cut-off values of body composition in this population owing to a limited sample size. Since the relatively lower BMI in East Asian patients may have a different prognostic impact, further studies are needed to explore the different strata of body composition. Third, ensuring exact similarities in the abdominal anatomy between the two selected 2D CT images from the preoperative and postchemotherapy phases is a particular challenge. Compared to skeletal muscle or subcutaneous fat, measurements of visceral fat may be particularly influenced by the voluntary movements of the digestive tract. This may have been reflected by the wider range of visceral fat changes in our study; however, we excluded patients with evidence of intestinal obstruction on preoperative CT scans to reduce the possibility of incorrect visceral fat measurements. The limitations of manually measured 2D image-based body composition may be overcome by using a 3D image-based measurement with machine learning algorithms [44]. Finally, weight loss during chemotherapy was previously reported to be a poor prognostic factor [45]. Visceral fat or muscle loss may translate to loss of body weight; a study of the correlation between these parameters would be of particular interest. In our study, body weight at the postchemotherapy stage was not fully recorded in entire cases; this was particularly uninformative. Further studies focusing on these relationships are warranted. 

## 4. Materials and Methods

This study was conducted after approval of the Institutional Review Board of the Gangnam Severance Hospital, Yonsei University College of Medicine (Seoul, Korea) (approval no; 3-2017-0099). The need for informed consent was waived in view of its retrospective design. 

Patients with stage III and high-risk stage II colon cancer who underwent curative surgery followed by FOLFOX chemotherapy between October 2005 and June 2013 were selected. The chemotherapy schedule given every two weeks was oxaliplatin (85 mg/m^2^ IV infusion of more than 2 h on day 1), leucovorin (400 mg/m^2^ IV infusion of more than 2 h on day 1), and 5-FU (400 mg/m^2^ IV bolus injection on day 1, followed by 2400 mg/m^2^ continuous IV injection over 46 h). A total of 12 cycles were planned. Patients who underwent at least one cycle of FOLFOX were initially selected in this study. Inclusion criteria were patients who had both preoperative and postchemotherapy CT scan results, body composition data available from the CT scans, and available clinicopathologic and follow-up data. Exclusion criteria were those who showed intestinal obstruction on the preoperative CT scans, underwent non-contrast CT alone, received neoadjuvant chemotherapy, and had a history of inflammatory bowel disease. 

### 4.1. Measured Outcomes of CT-based Anthropometric Values

In our hospital, abdominopelvic CT was performed to evaluate the presence of systemic metastases before surgery and within 1 month after completion of planned adjuvant chemotherapy. Most of the enrolled patients underwent postchemotherapy CT after completion of planned chemotherapy. However, the CT scanning was performed earlier than planned in certain patients with elevated levels of carcinoembryonic antigen during chemotherapy; this was necessary to detect any potential systemic metastases. 

A single cross-sectional transverse CT image at the L3 vertebra level was selected for analysis. The portal phase image was initially selected; in cases where it was not available, arterial phase images were selected to measure the body composition. For evaluation of skeletal muscle, visceral fat, and subcutaneous fat, this cross-sectional image was entered into the in-house open-source software named BMI_CT, which was based on the MATLAB Complier (R2014a) Runtime 8.3 (Mathworks Inc., Natick, MA, USA) software package [46]. This program is available online (https://sourceforge.net/projects/muscle-fat-area-measurement/; final access date: 30 September 2019) and has been used to measure body composition in a previous study [47]. In addition, the radiodensity of skeletal muscle was defined as the mean value of selected skeletal muscle and was measured using open-source three-dimensional (3D) slicer software (https://slicer.org/) [48]. A randomly selected sub-sample of 30 CT images was analyzed by two investigators blinded to the clinical outcomes, to calculate the intra-class correlation coefficients (Supplementary Table S3. The remaining CT images were analyzed by a trained physician. 

The skeletal muscle, visceral fat, subcutaneous fat, and SMR were initially measured. The total fat was defined as the summation of visceral and subcutaneous fat. The skeletal muscle area was calculated based on the Hounsfield unit (HU) thresholds of −29 and 150. Subcutaneous fat was calculated based on HU thresholds between −190 and −30, whereas visceral fat was calculated based on a radiodensity ranging between −150 and −50 HU. The SMI, VFI, SFI (subcutaneous fat index), and TFI were calculated by dividing each variable by the square of the patient’s height (cm^2^/m^2^). 

The variables measured from preoperative CT scans were named SMI_pre, SMR_pre, VFI_pre, SFI_pre, and TFI_pre. The variables measured using postchemotherapy CT scans were defined as SMI_post, SMR_post, VFI_post, SFI_post, and TFI_post. 

**Figure 2:**
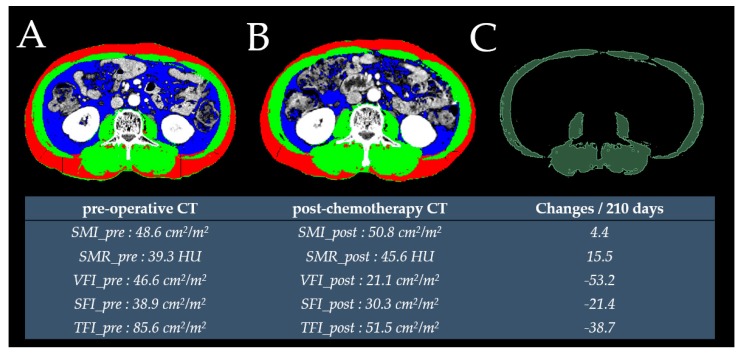
Cross-sectional L3 CT image used to measure subcutaneous fat (red), skeletal muscle (green) and visceral fat (blue) using preoperative (**A**) and postchemotherapy CT (**B**) images. The radiodensity of skeletal muscle in the preoperative CT was measured using an open-source three-dimensional slicer software (**C**).

The median duration between preoperative CT and surgery was 8 (interquartile range [IQR]: 5–12.5) days. The median duration between preoperative and postchemotherapy CT scans was 217 (IQR: 200.5–231.5) days. The actual change in each variable was divided by the number of days between CT examinations and multiplied by 210 to account for variations in the durations of scan intervals. Therefore, the SMIC, SMRC, VFIC, SFIC, and TFIC were defined as follows: (postchemotherapy value − preoperative value)/preoperative value × 100/(days between postchemotherapy and preoperative CT scans) × 210 (Figure 2).

### 4.2. Defining the Cut-Off Values

Among the parameters measured using preoperative CT and postchemotherapy CT, low SMI and low SMR were defined as the recommended values based on the study by Martin and colleagues [49]. The VFI, SFI, and TFI were divided using values of 52.9 cm^2^/m^2^ in males and 51.5 cm^2^/m^2^ in females, 50 cm^2^/m^2^ in males and 42 cm^2^/m^2^ in females, and 107.7 cm^2^/m^2^ in males and 102.2 cm^2^/m^2^ in females, respectively; these values were derived from a previous study (Supplementary Table S4 [50]).

Changes in the variables (SMIC, SMRC, VFIC, SFIC, and TFIC) were also considered. The cut-off values for SMIC and SMRC were set at −2%; considering the potential 2% measurement error range, these subgroups were defined as “loss of 2% or more” versus “loss of less than 2%” for SMIC and SMRC, respectively. Owing to the absence of reliable recommendations for changes in fat area, the optimal cut-off values for VFIC, SFIC, and TFIC were defined as the values that provided the largest χ2 on the Mantel–Cox test and were defined by the X-tile program (Version 3.6.1, Yale University School of Medicine, New Haven, CT, USA) [51]. The dichotomized groups were presented based on “loss of cut-off point or more” and “loss of less than cut-off point.” 

### 4.3. Statistical Analysis

Patients’ clinicopathological characteristics were compared using the chi-square and *t*-tests for categorical and continuous variables, respectively. The paired *t*-test and Wilcoxon signed-rank test were used to assess changes in body composition. Spearman’s correlation coefficient was used to assess relationships between continuous variables. 

Overall survival (OS) was calculated from the date of surgery to the date of death or the last known date of being alive. The Kaplan–Meier method with log-rank tests was used to construct the survival curves. Cox proportional hazards models were used to estimate the hazard ratios (HRs) and 95% confidence interval (CI). All variables with a *p* < 0.1 on univariable analysis were entered for multivariable analysis. Owing to multicollinearity, we performed forward stepwise selection of variables based on the Akaike information criterion. A two-sided *p* < 0.05 was considered statistically significant. All statistical analyses were performed using SPSS software, version 23.0 (SPSS, Chicago, IL, USA) and R version 3.5.1 (R-project, Institute for Statistics and Mathematics, Vienna, Austria).

## 5. Conclusions

In conclusion, in patients with non-metastatic colon cancer who underwent FOLFOX adjuvant chemotherapy, our study demonstrated that severe visceral fat loss and myosteatosis detected during the postchemotherapy period could be used to stratify patients at high-risk of mortality. Further studies are needed to investigate whether a reversal of these changes may improve survival. Our findings represent a novel association between body composition and patient outcomes and suggest that changes in composition may be used as indicators during surveillance. Further prospective studies in larger cohorts are needed to validate our findings.

## Figures and Tables

**Table 1:** Comparison of patient characteristics and clinicopathological outcomes according to gender.

Variables		Female (*n* = 67) (%)	Male (*n* = 100) (%)	*p*
Age (years)	<65	51 (76.1)	63 (63)	0.106
	≥65	16 (23.9)	37 (37)	
ASA grade	I	32 (47.8)	43 (43)	0.928
	II	25 (37.3)	39 (39)	
	III	5 (7.5)	9 (9)	
	No data	5 (7.5)	9 (9)	
BMI (kg/m^2^)	<25	55 (82.1)	66 (66)	0.035
	≥25	12 (17.9)	34 (34)	
Smoking	Yes	0	47 (47)	<0.001 ^a^
	No	67 (100)	53 (53)	
CEA (ng/mL)	<5	47 (70.1)	63 (63)	0.430
	≥5	20 (29.9)	37 (37)	
Tumor location ^b^	proximal	28 (41.8)	31 (31)	0.206
	distal	39 (58.2)	69 (69)	
Operation time (min)	<300	50 (74.6)	84 (84)	0.196
	≥300	17 (25.4)	16 (16)	
Complications	No	59 (88.1)	82 (82)	0.4
	Yes	8 (11.9)	18 (18)	
Tumor size (cm)	<5	39 (58.2)	63 (63)	0.645
	≥5	28 (41.8)	37 (37)	
LVI	Absent	37 (55.2)	60 (60)	0.753 ^a^
	Present	26 (38.8)	36 (36)	
	No data	4 (6)	4 (4)	
Stage	II	10 (14.9)	16 (16)	>0.99
	III	57 (85.1)	84 (84)	
Chemotherapy cycles	<10	8 (11.9)	14 (14)	0.879
	≥10	59 (88.1)	86 (86)	

Abbreviations—BMI: body mass index; ASA: American Society of Anesthesiologists; CEA: carcinoembryonic antigen; LVI: lymphovascular invasion. ^a^ Fisher’s exact test. ^b^ Tumor location—proximal: cecum-transverse colon; distal: descending colon-rectosigmoid junction. Two patients with synchronous colon cancer were classified into the distal group for statistical reasons.

**Table 2:** Comparison of preoperative and postchemotherapy values, and changes of anthropometric values according to gender.

	Female (*n* = 67) (Mean ± SD)	Male (*n* = 100) (Mean ± SD)	*p* ^a^
**Values at preoperative CT**			
SMI_pre (cm^2^/m^2^)	38.9 ± 5.7	47.9 ± 7.5	<0.001
SMR_pre (HU)	41.2 ± 8.8	44.4 ± 7.4	0.012
VFI_pre (cm^2^/m^2^)	30.1 ± 22.6	41.1 ± 23.8	0.003
SFI_pre (cm^2^/m^2^)	56.0 ± 23.9	36.2 ± 15.6	<0.001
TFI_pre (cm^2^/m^2^)	86.1 ± 41	77.3 ± 37.2	0.153
**Values at postchemotherapy CT**			
SMI_post (cm^2^/m^2^)	43.5 ± 5.4	51.6 ± 8.1	<0.001
SMR_post (HU)	42.1 ± 7.5	46.5 ± 6.7	<0.001
VFI_post (cm^2^/m^2^)	24.8 ± 17.1	30.3 ± 17.6	0.048
SFI_post (cm^2^/m^2^)	56.2 ± 22.5	36.2 ± 15.0	<0.001
TFI_post (cm^2^/m^2^)	81.0 ± 35.1	66.5 ± 30.7	0.005
**Percentage changes over 210 days between preoperative and postchemotherapy CTs**			
SMIC (%/210 days)	12.8 ± 12.7	8.6 ± 12.0	0.030
SMRC (HU/210 days)	4.1 ± 18.1	5.7 ± 14.5	0.560
VFIC (%/210 days)	12.6 ± 80.9	5.6 ± 124.5	0.660
SFIC (%/210 days)	7.6 ± 33.2	7.3 ± 44.9	0.955
TFIC (%/210 days)	3.6 ± 36.9	−1.0 ± 58.4	0.541

^a^ This comparison was done between values from the female group and values from the male group separately.

**Table 3:** Distribution of the percentage changes in the skeletal muscle and fat tissues over 210 days during adjuvant FOLFOX chemotherapy in patients with non-metastatic colon cancer (*n* = 167).

Value Distribution	SMIC	SMRC	VFIC	SFIC	TFIC
Minimum	−21.3	−34.29	−95.33	−68.94	−76.33
25th percentile	2.75	−4.4	−40.68	−16.04	−24.66
Median	8.7	3.42	−19.04	−3.46	−11.93
75th percentile	17.05	13.40	8.67	19.78	10.33
Maximum	59.8	56.43	648.39	172.59	260.48

**Table 4:** Summary of the Kaplan–Meier curve comparison of overall survival according to the dichotomized groups.

Preoperative CT		Postchemotherapy CT		Changes/210 Days between Two CTs	
SMI_pre low vs. SMI_pre high	*p* = 0.8	SMI_post low vs. SMI_post high	*p* = 0.4	SMIC: Loss of 2% or more vs. Loss of less than 2%	*p* = 0.16
SMR_pre low vs. SMR_pre high	*p* = 0.066	SMR_post low vs. SMR_post high	*p* = 0.05	SMRC: Loss of 2% or more vs. Loss of less than 2%	*p* = 0.94
VFI_pre low vs. VFI_pre high	*p* = 0.28	VFI_post low vs. VFI_post high	*p* = 0.89	VFIC: Loss of 46.57% or more vs. Loss of less than 46.57%	*p* = 0.00078
SFI_pre low vs. SFI_pre high	*p* = 0.45	SFI_post low vs. SFI_post high	*p* = 0.37	SFIC: Loss of 17.03% or more vs. Loss of less than 17.03%	*p* = 0.091
TFI_pre low vs. TFI_pre high	*p* = 0.74	TFI_post low vs. TFI_post high	*p* = 0.22	TFIC: Loss of 42.61% or more vs. Loss of less than 42.61%	*p* = 0.0033

**Table 5:** Univariable analysis for overall survival.

		Univariable Analysis	
Variables		Hazard Ratio (95% CI)	*p*
Gender	Female	1	
	Male	1.79 (0.83–3.88)	0.137
Age (years)	<65	1	
	≥65	0.76 (0.35–1.65)	0.492
ASA grade	1	1	
	2	0.77 (0.35–1.70)	0.524
	3	0.75 (0.17–3.31)	0.715
	No data	1.02 (0.33–3.11)	0.965
BMI (kg/m^2^)	<25	1	
	≥25	0.59 (0.24–1.44)	0.253
Smoking	No	1	
	Yes	1.83 (0.90–3.72)	0.091
CEA (ng/mL)	<5	1	
	≥5	1.12 (0.55–2.3)	0.743
Tumor location ^a^	proximal	1	
	distal	0.64 (0.32–1.29)	0.219
Operation time (min)	<300	1	
	≥300	2.59 (1.25–5.38)	0.01
Complications	No	1	
	Yes	1.75 (0.78–3.9)	0.169
Tumor size (cm)	<5	1	
	≥5	1.04 (0.51–2.11)	0.912
LVI	Absent	1	
	Present	2.21 (1.07–4.57)	0.03
	No data	1.58 (0.35–7.07)	0.545
Stage	II	1	
	III	1.54 (0.53–4.40)	0.419
Chemotherapy cycles	<10	1	
	≥10	0.47 (0.20–1.09)	0.08
SMI_pre	Low	1	
	High	0.91 (0.45–1.82)	0.799
SMR_pre	Low	1	
	High	0.52 (0.25–1.05)	0.070
VFI_pre	Low	1	
	High	1.5 (0.71–3.18)	0.284
SFI_pre	Low	1	
	High	0.75 (0.36–1.57)	0.457
TFI_pre	Low	1	
	High	1.13 (0.53–2.39)	0.746
SMI_post	Low	1	
	High	0.73 (0.35–1.51)	0.401
SMR_post	Low	1	
	High	0.48 (0.23–1.01)	0.055
VFI_post	Low	1	
	High	0.91 (0.27–3.01)	0.887
SFI_post	Low	1	
	High	0.71 (0.34–1.48)	0.372
TFI_post	Low	1	
	High	0.48 (0.14–1.58)	0.229
SMIC	Loss of 2% or more	1	
	Loss of less than 2%	0.54 (0.23–1.27)	0.162
SMRC	Loss of 2% or more	1	
	Loss of less than 2%	0.97 (0.46–2.01)	0.939
VFIC	Loss of 46.57% or more	1	
	Loss of less than 46.57%	0.31 (0.15–0.64)	0.001
SFIC	Loss of 17.03% or more	1	
	Loss of less than 17.03%	0.53 (0.25–1.17)	0.096
TFIC	Loss of 42.61% or more	1	
	Loss of less than 42.61%	0.31 (0.14–0.71)	0.005

Abbreviations—BMI: body mass index; ASA: American Society of Anesthesiologists; CEA: carcinoembryonic antigen; LVI: lymphovascular invasion; CI: confidence interval. ^a^ Tumor location—proximal: cecum-transverse colon; distal: descending colon-rectosigmoid junction. Two patients with synchronous colon cancer were classified into the distal group for statistical reasons.

**Table 6:** Multivariable analysis for overall survival.

		Multivariable Analysis	
Variables		Hazard Ratio (95% CI)	*p*
Smoking	No	1	
	Yes	2.25 (1.06–4.77)	0.034
Operation time (min)	<300	1	
	≥300	3.87 (1.80–8.33)	0.0005
LVI	Absent	1	
	Present	2.46 (1.16–5.18)	0.017
	No data	1.19 (0.26–5.39)	0.818
SMR_post	Low	1	
	High	0.32 (0.15–0.70)	0.004
VFIC	Loss of 46.57% or more	1	
	Loss of less than 46.57%	0.31 (0.14–0.69)	0.004
SFIC	Loss of 17.03% or more	1	
	Loss of less than 17.03%	0.53 (0.23–1.19)	0.128

Abbreviations: LVI: lymphovascular invasion; CI: confidence interval; Factors with p value less than 0.1 in univariable analysis were entered into multivariable analysis.

## Supplementary Materials

The following are available online at https://www.mdpi.com/2072-6694/12/1/60/s1, Table S1. Absolute changes in body composition during FOLFOX adjuvant chemotherapy; Table S2. Correlation coefficient of changes in body composition during FOLFOX adjuvant chemotherapy; Table S3. Intraclass correlation coefficients according to the body compositions (n = 30); Table S4. Cut-off values from previous studies adopted in this study; Figure S1. Distribution of number of completed cycles of FOLFOX chemotherapy; Figure S2. Distribution of the percentage changes in body composition over 210 days (n = 167); Figure S3. Kaplan–Meier curve comparison of overall survival according to the dichotomized anthropometric variables measured at the preoperative CT (n = 167); Figure S4. Kaplan–Meier curve comparison of overall survival according to the dichotomized anthropometric variables measured at the postchemotherapy CT (n = 167); Figure S5. Kaplan–Meier curve comparison of overall survival according to the dichotomized changes of anthropometric variables during chemotherapy (n = 167).
