# Supplementary material for: Changes in Body Composition during Adjuvant FOLFOX Chemotherapy and Overall Survival in Non-Metastatic Colon Cancer
Source: Cancers (Basel). 2019 Dec 24;12(1):60. doi: 10.3390/cancers12010060 (PMC7016804; doi:10.3390/cancers12010060)
Supplement: Supplementary file 1 [file cancers-12-00060-s001.pdf]

cancers

Supplementary

Changes in Body Composition During Adjuvant FOLFOX Chemotherapy and Overall Survival in Non-metastatic Colon Cancer

Figure S1. Distribution of number of completed cycles of FOLFOX chemotherapy.

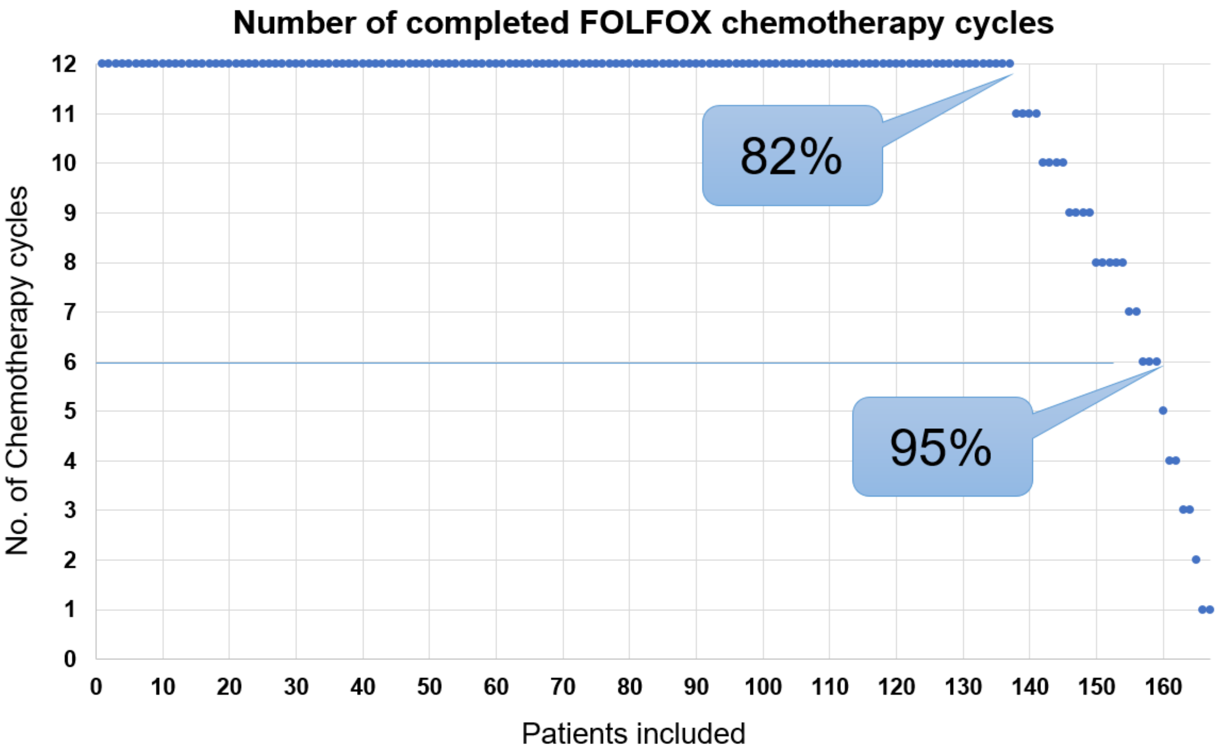

: 82% of patients completed the total 12 cycle of FOLFOX chemotherapy and 95% of patients completed more than 6 cycles of planned FOLFOX chemotherapy.

Figure S2. Distribution of the percentage changes in body compositions over 210 days (n=167).

A. Change in skeletal muscle index over 210 days

B. Change in skeletal muscle radiodensity over 210 days

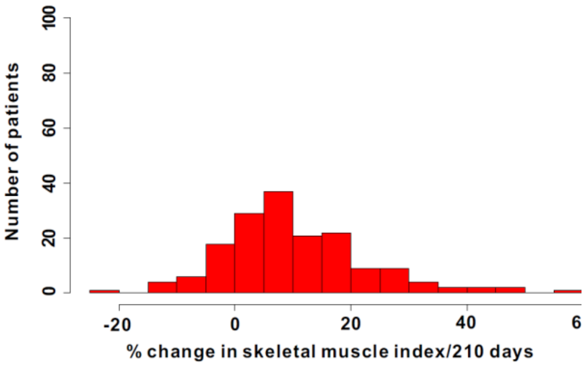

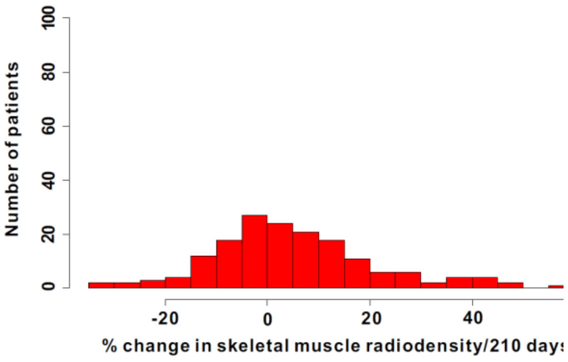

C. Change in visceral fat index over 210 days

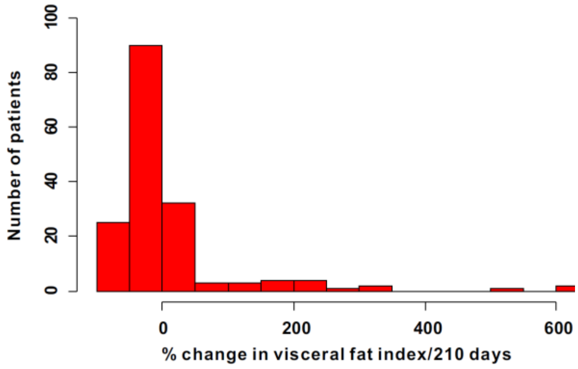

D. Change in subcutaneous fat index over 210 days

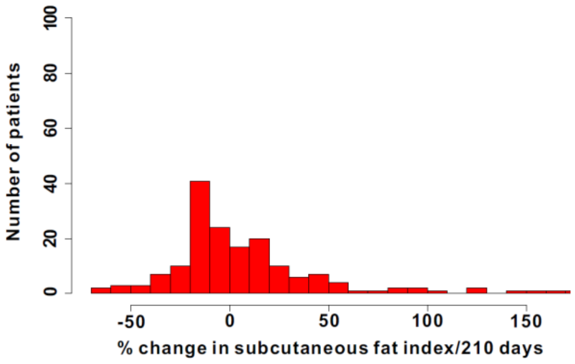

E. Change in total fat index over 210 days

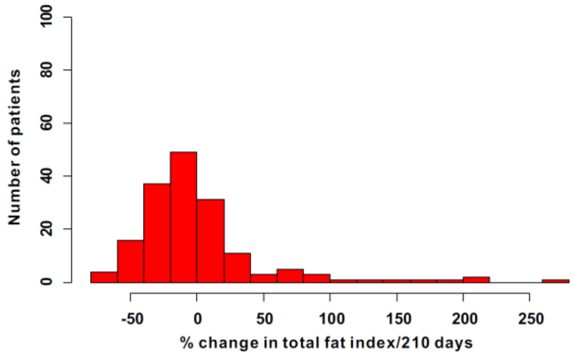

Figure S3. Kaplan-Meier curve comparison of overall survival according to the dichotomized anthropometric variables measured at the pre-operative CT (n=167).

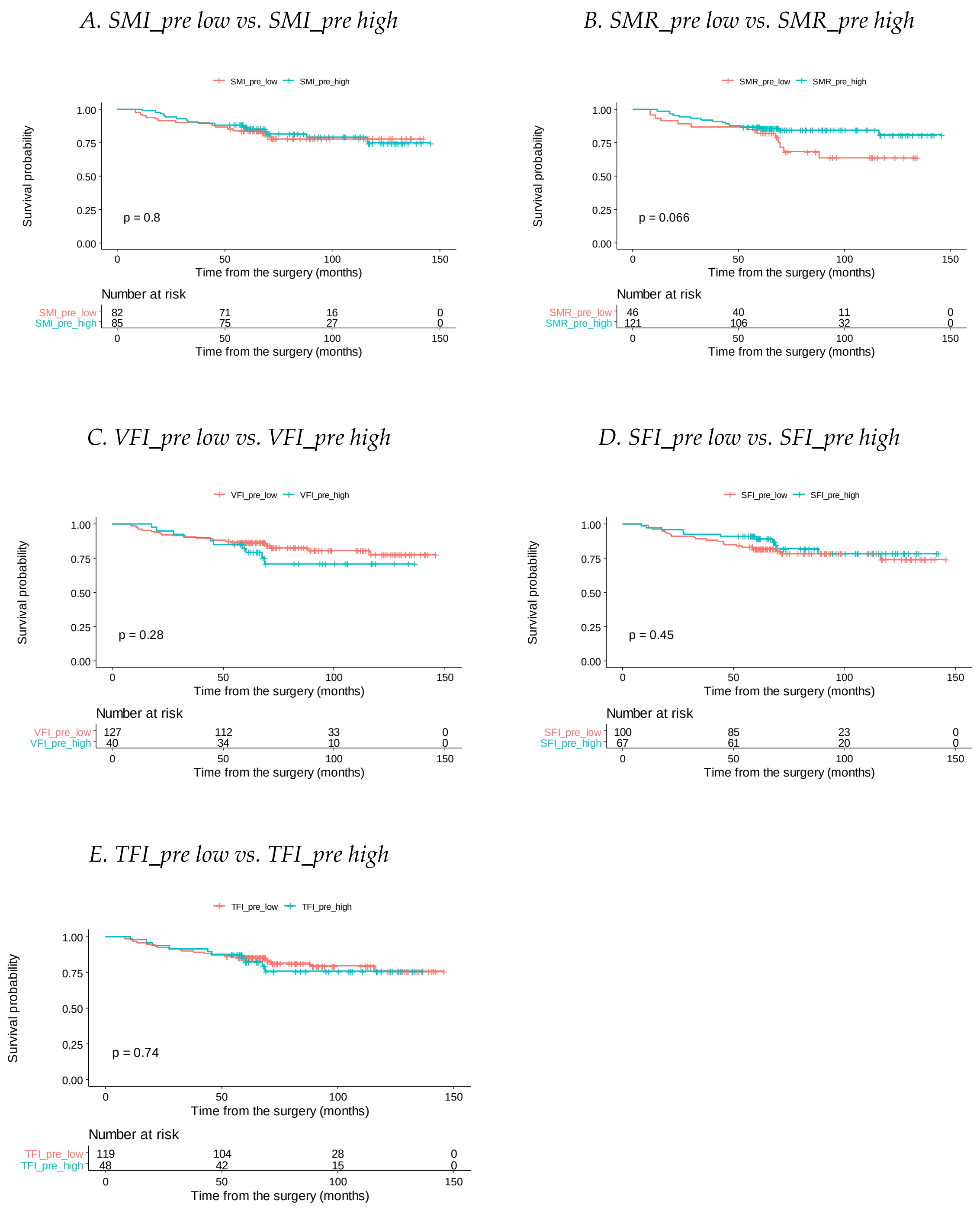

Figure S4. Kaplan-Meier curve comparison of overall survival according to the dichotomized anthropometric variables measured at the post-chemotherapy CT (n=167).

A. SMI_post low vs. SMI_post high

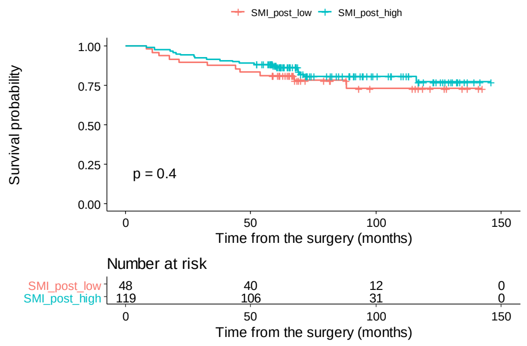

B. SMR_post low vs. SMR_post high

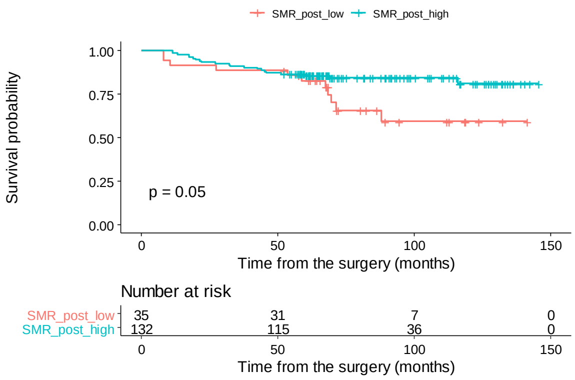

C. VFI_post low vs. VFI_post high

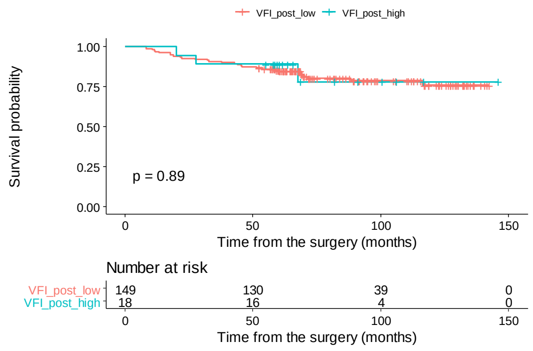

D. SFI_post low vs. SFI_post high

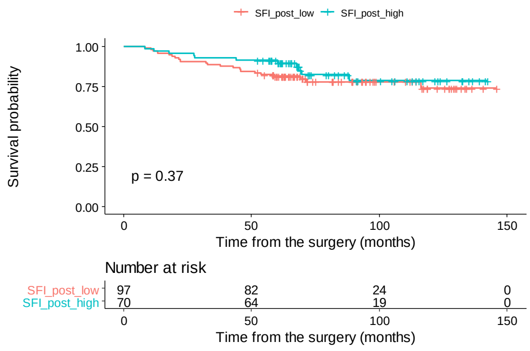

E. TFI_post low vs. TFI_post high

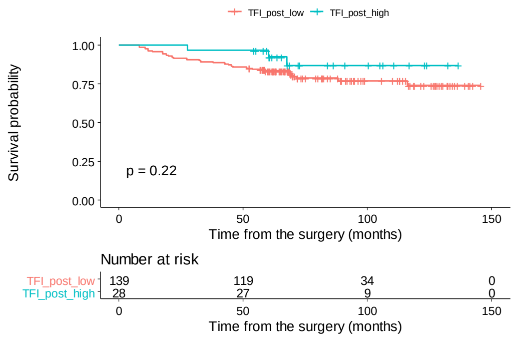

Figure S5. Kaplan-Meier curve comparison of overall survival according to the dichotomized changes of anthropometric variables during chemotherapy (n=167).

A. SMIC : Loss of 2% or more vs. Loss of less than 2%

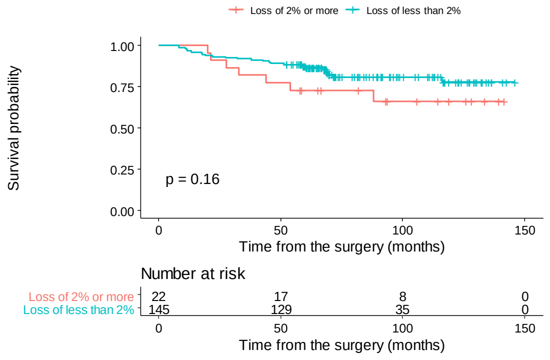

B. SMRC : Loss of 2% or more vs. Loss of less than 2%

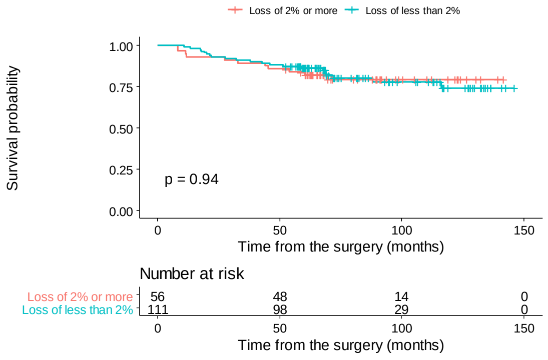

C. VFIC : Loss of 46.57% or more vs. Loss of less than 46.57%

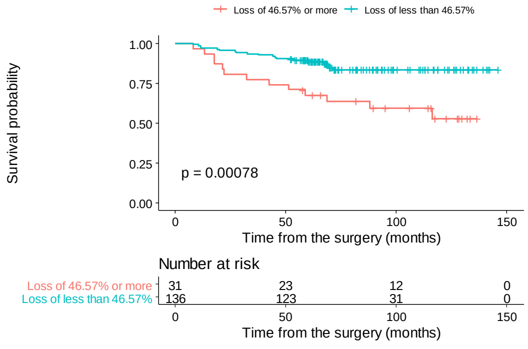

D. SFIC: Loss of 17.03% or more vs. Loss of less than 17.03%

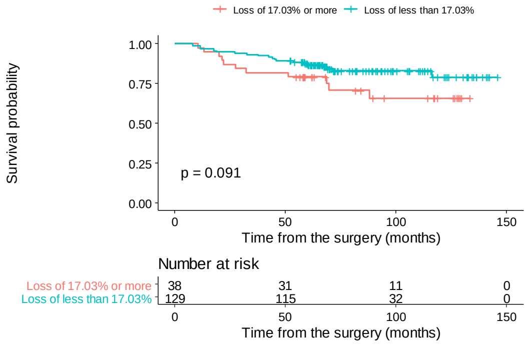

E. TFIC : Loss of 42.61% or more vs. Loss of less than 42.61%

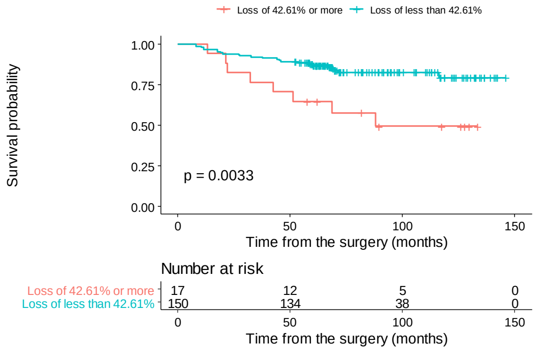

Table S1. Absolute changes in body composition during FOLFOX adjuvant chemotherapy.

	SMI	SMR	VFI	SFI	TFI
Shapiro-Wilk test	$p = 0.004$	$p = 0.685$	$p < 0.001$	$p = 0.025$	$p = 0.018$
Values_pre-operative					
(mean ± SD)	44.2 ± 8.1	43.1 ± 8.1	36.7 ± 23.8	44.1 ± 21.6	80.8 ± 38.9
Values_post-chemotherapy					
(mean ± SD)	48.3 ± 8.1	44.7 ± 7.3	28.0 ± 17.5	44.1 ± 20.7	72.2 ± 33.1
Paired differences (mean ± SD)	-4.1 ± 4.9	-1.6 ± 6.1	8.6 ± 16.8	-0.0 ± 12.4	8.55 ± 25.0
95%CI	-4.8 / -3.3	-2.5 / -0.6	6.0 / 11.2	-1.9 / 1.8	4.7 / 12.3
Paired t test	NA	$p = 0.001$	NA	NA	NA
Wilcoxon signed rank test	$p < 0.001$	NA	$p < 0.001$	$p = 0.936$	$p < 0.001$

SD: Standard Deviation; CI: Confidence Interval; NA: Not Available.

Table S2. Correlation coefficient of changes in body composition during FOLFOX adjuvant chemotherapy.

	SMI	SMR	VFI	SFI	TFI
Paired samples correlations :					
Correlation efficient	0.817	0.683	0.709	0.828	0.769
P value	$p < 0.001$	$p < 0.001$	$p < 0.001$	$p < 0.001$	$p < 0.001$

Table S3. Intraclass correlation coefficients according to the body compositions (n=30).

Variables	ICC	low	upper
Skeletal muscle area	0.97	0.95	0.99
Skeletal muscle radiodensity	0.99	0.97	0.99
Visceral fat area	0.99	0.97	0.99
Subcutaneous fat area	1	0.99	1

Table S4. Cut-off values from previous studies adopted in this study.

	SMI (cm^2/m^2)	SMR (HU)	VFI (cm^2/m^2)	SFI (cm^2/m^2)	TFI (cm^2/m^2)
Reference	Martin et al.[1]		Ebadi M est al.[2]		
Male	43 (BMI<25 kg/m^2), 53 (BMI ≥25 kg/m^2)	41 (BMI<25 kg/m^2), 33 (BMI ≥25 kg/m^2)	52.9	50	107.7
Female	41	41 (BMI<25 kg/m^2), 33 (BMI ≥25 kg/m^2)	51.5	42	102.2

References

1. Martin L, Birdsell L, Macdonald N, Reiman T, Clandinin MT, McCargar LJ, et al. Cancer cachexia in the age of obesity: skeletal muscle depletion is a powerful prognostic factor, independent of body mass index. *J Clin Oncol* 2013;**31**:1539-1547.
2. Ebadi M, Martin L, Ghosh S, Field CJ, Lehner R, Baracos VE, et al. Subcutaneous adiposity is an independent predictor of mortality in cancer patients. *Br J Cancer* 2017;**117**:148-155.
